# Pandora’s Box–Influence of Contour Parameters on Roughness and Subsurface Residual Stresses in Laser Powder Bed Fusion of Ti-6Al-4V

**DOI:** 10.3390/ma13153348

**Published:** 2020-07-28

**Authors:** Katia Artzt, Tatiana Mishurova, Peter-Philipp Bauer, Joachim Gussone, Pere Barriobero-Vila, Sergei Evsevleev, Giovanni Bruno, Guillermo Requena, Jan Haubrich

**Affiliations:** 1 Institute of Materials Research, German Aerospace Center (DLR; Deutsches Zentrum für Luft-und Raumfahrt), Linder Höhe, 51147 Cologne, Germany; Peter-Philipp.Bauer@dlr.de (P.-P.B.); joachim.gussone@dlr.de (J.G.); pere.barrioberovila@dlr.de (P.B.-V.); guillermo.requena@dlr.de (G.R.); jan.haubrich@dlr.de (J.H.); 2 Bundesanstalt für Materialforschung und–Prüfung (BAM; Federal Institute for Materials Research and Testing), Unter den Eichen 87, 12205 Berlin, Germany; tatiana.mishurova@bam.de (T.M.); sergei.evsevleev@bam.de (S.E.); giovanni.bruno@bam.de (G.B.); 3 Institute of Physics and Astronomy, University of Potsdam, Karl-Liebknecht-Straße 24/25, 14476 Potsdam, Germany; 4 Metallic Structures and Materials Systems for Aerospace Engineering, RWTH Aachen University, 52062 Aachen, Germany

**Keywords:** Ti-6Al-4V, additive manufacturing, contour scan strategy, surface roughness, melt pool monitoring, residual stress, synchrotron X-ray diffraction

## Abstract

The contour scan strategies in laser powder bed fusion (LPBF) of Ti-6Al-4V were studied at the coupon level. These scan strategies determined the surface qualities and subsurface residual stresses. The correlations to these properties were identified for an optimization of the LPBF processing. The surface roughness and the residual stresses in build direction were linked: combining high laser power and high scan velocities with at least two contour lines substantially reduced the surface roughness, expressed by the arithmetic mean height, from values as high as 30 µm to 13 µm, while the residual stresses rose from ~340 to about 800 MPa. At this stress level, manufactured rocket fuel injector components evidenced macroscopic cracking. A scan strategy completing the contour region at 100 W and 1050 mm/s is recommended as a compromise between residual stresses (625 MPa) and surface quality (14.2 µm). The LPBF builds were monitored with an in-line twin-photodiode-based melt pool monitoring (MPM) system, which revealed a correlation between the intensity quotient I_2_/I_1_, the surface roughness, and the residual stresses. Thus, this MPM system can provide a predictive estimate of the surface quality of the samples and resulting residual stresses in the material generated during LPBF.

## 1. Introduction

Additive manufacturing (AM) technologies like directed energy deposition (DED) or laser powder bed fusion (LPBF) have been developed for the near-net-shape fabrication of metal components with high material efficiencies [1,2]. However, due to the nature of the processes and the used feedstocks (e.g., powders or wires), the obtainable geometric accuracies and surface qualities vary considerably [3] and are generally inferior to subtractive machining.

The process parameters and processing strategies employed in LPBF determine the contour properties significantly. This is mostly due to powder particles remaining attached to the resolidified outer surfaces (i.e., secondary roughness), stair-case effects due to discrete layer-wise processing, the melt tracks, and, thus, the contour laser process itself (primary roughness or waviness) [4,5,6,7]. The surface roughness depends strongly on the build angle of the surface, e.g., a side, down- or up-skin surface [5,8]. This has a high impact on the fatigue properties of LPBF coupons or components [4,7,9]. In contrast, pores and defects in the bulk or even near-surface play a much smaller role for crack initiation and fatigue life [4]. Particularly, the combination of poor surface quality and high tensile stresses is reported to be critical in an as-built condition of LPBF Ti-6Al-4V, whereas in specimens with high compressive residual stresses (i.e., after hot isostatic pressing and subsequent shot-peening), a high surface roughness (of R_a_ ≤ 21 µm) plays a negligible role on crack initiation [8]. Leuders et al. concluded that “any improvement of surface roughness may greatly improve the cyclic properties” [8]. Since the primary roughness is one critical factor responsible for the degradation of the fatigue properties of LPBF Ti-6Al-4V [4], the optimization of the LPBF contour strategy and laser parameters is of major importance for the near-net-shape manufacturing of highly loaded components without expensive and time-consuming surface post-processing.

The scan strategies and laser parameters in LPBF also strongly influence the formation of residual stresses (RSs) [10,11,12,13,14,15,16]. The origins of the RS in AM are the steep and local thermal gradients developing during melting, remelting, reheating, solidification, and cool-down (see further Refs. [9,10,17]). The RSs in the as-built state prior to any heat treatment are generally sizable (up to ~800 MPa in Ti alloys) and may lead to detachment of support structures, cracking of parts, or geometrical distortion during manufacturing [8,18,19]. They are tensile in nature at the surface and in subsurface regions [16,20] and are balanced by compressive stresses in the bulk of LPBF materials [21]. Therefore, every scan and laser parameter in LPBF, including those in the contour region, influences the thermal history of the part and the RS. Especially, different scan strategies have been studied by various research groups. While some reported rotating scan patterns achieved more homogeneous temperature distributions, which result in low RS [10,15], others concluded that stripe patterns alternating by 90° minimized RS [11,14].

The high tensile RS can be relieved by heat post-treatment (e.g., stress-relieving treatments; see Ref. [22] and therein) and even transformed to compressive stresses by post-processing by, e.g., laser or shot peening [8]. Generally, a stress relieve heat treatment of LPBF Ti-6Al-4V in a temperature range between 600 and 700 °C for times of ca. 2 to 3 h typically is sufficient to quantitatively eliminate the tensile stresses [22,23]. This is generally carried out prior to the removal of LPBF components from the build plates in order to avoid part distortion. Besides the reduction of RS, this thermal treatment promotes the decomposition of martensitic microstructures, which are present in the LPBF Ti-6Al-4V as-built conditions. This process leads to improved trade-offs between strength and ductility [24,25,26].

For advancing the technological maturity of metal AM, current research efforts focus also on process monitoring and quality control for fast and cost-effective solutions. With in situ monitoring systems, a better process understanding, higher reproducibility, and part quality might be achieved. In the long-term, a feedback-control system with a “self-optimization of the manufacturing process” [27] may be attainable. Several types of in situ monitoring systems for AM machines are already commercially available [28,29,30,31]. Most of these systems are based on optical methods [32,33,34,35,36,37,38,39,40], consisting of spatially resolved sensors (e.g., cameras) or spatially integrating sensors (i.e., pyrometers/photodiodes). An overview of melt pool monitoring (MPM) results is given, e.g., in [41,42,43,44,45].

Despite the growing number of experimental and simulation studies on the formation of residual stresses in LPBF of titanium alloys (e.g., [8,9,11,12,13,15,16,17,18,21]), the role of contour strategies for tackling subsurface residual stresses, as well as their interplay with the surface qualities, has received little attention so far. Hence, the present study was conducted to provide new insights for the optimization of contour scan strategies and contour laser parameters based on surface qualities and subsurface residual stress formation. Extensive use of the integrated melt pool monitoring tool developed by SLM Solutions GmbH was made for in situ process monitoring in order to study its potential as a tool to assess and estimate these properties prior to any part analysis. Besides proposing an optimized LPBF strategy for contour processing, a correlation between the intensity quotient I_2_/I_1_, the surface roughness, and the residual stresses was found, enabling a prediction of these properties from MPM already during a build. Surface roughness and the residual stresses were affected in an opposing manner by the contour parameters, requiring that any optimized or balanced contour scan strategy leads to a compromise between both properties.

## 2. Materials and Methods

### 2.1. Sample Manufacturing

All samples were manufactured with an SLM 280^HL^ from SLM Solutions GmbH (Luebeck, Germany) with an integrated melt pool monitoring system. For the programming of the build job files, the software Magics (version 21.1, Materialise, Leuven, Belgium) and the SLM Solutions Metal build processor were used. The build platform was pre-heated to 200 °C, and the LPBF process was carried out in inert argon gas (purity 5.0).

**Figure 1:**
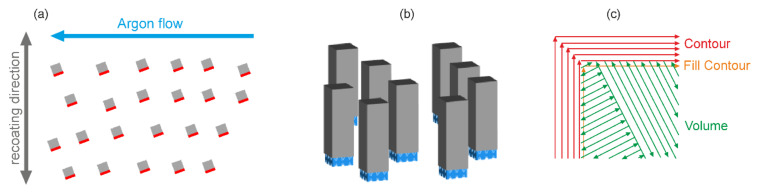
(**a**) Sample positions on the base plate; red marked are the sample sides, which were investigated in this study; (**b**) side view of the specimens (5 × 5 × 15 mm^3^), which were printed on 2 mm block supports; (**c**) example for a typical scan pattern used in this study consisting of 5 contour lines, 1 fill contour, and volume scan.

Plasma-atomized Ti-6Al-4V ELI grade 23 powder with a particle size of d_90_ < 50 μm (AP&C, Boisbriand Québec, Canada) was used. All specimens (dimensions 5 × 5 × 15 mm^3^) were built on 2 mm tall block supports (Figure 1). The specimens were systematically positioned close to each other (approx. 20 mm apart; Figure 1a) to reduce the influence of the specimens’ placement on the base plate on the resulting material properties [13]. In order to ensure a more homogeneous powder recoating process, all samples were rotated by 20° against the recoater direction. In the subsequent measurements, the same side surface on each specimen (marked in red in Figure 1a) was investigated to exclude side dependent effects [12].

The details of the process parameters that were studied are listed in Appendix A.

For the sake of an easy comparison, the descriptor volume energy density E_v_ has been used in this study with the laser power P, the scanning velocity v, the hatch distance h, and the layer thickness t (Equation (1)).

(1) $$E_{v}=\frac{P}{v\cdot h\cdot t}$$

**Figure 2:**
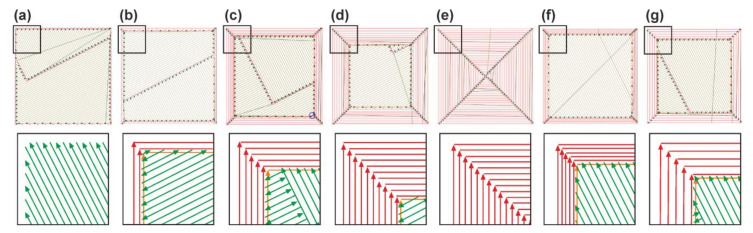
Examples for different scan patterns (bottom images represent enlargements of the top left corner in the upper sketches): (**a**) only chess pattern used (contour line with P = 0 W); (**b**) 2 contour lines (h = 90 µm); (**c**) 5 contour lines (h = 90 µm), (**d**) 10 contour lines (h = 90 µm); (**e**) only contour lines (h = 90 µm); (**f**) 5 contour lines with a small hatch distance of h = 60 µm; (**g**) 5 contour lines with a large hatch distance of h = 120 µm.

A layer thickness of t = 30 µm was chosen. For most of the specimens, two different parameter sets were used, one parameter set for the contour and fill contour and one for the volume (see example in Figure 1c). For the volume, a chess pattern with a minimum field size of 5 mm was used, which was rotated by 90° from layer to layer. The same laser parameters were applied to the contour lines and to the fill contour. The only difference between these two types of the contour is that the fill contour lies within the area of the volume and, hence, this area is remelted during the scan. The fill contour is often used to reduce porosity in the transition area between the volume hatching vectors and the outer contour. In order to study the influence of contour process parameters, the process parameters, as well as the scan order, were varied. Examples of different scan patterns are depicted in Figure 2.

After manufacturing, the specimens were removed carefully from the build plate without influencing the residual stress state. Finally, the excess powder was removed ultrasonically.

For comparison, a selected post-processing treatment was conducted for one of the specimens: The specimen was first grit-blasted (4 bar) and afterward shot-peened for 1 min with Zirshot 210 (Carlo Bernasconi AG, Bern, Switzerland) at 6 bar (sample distance: 50–60 mm).

### 2.2. Melt Pool Monitoring

**Figure 3:**
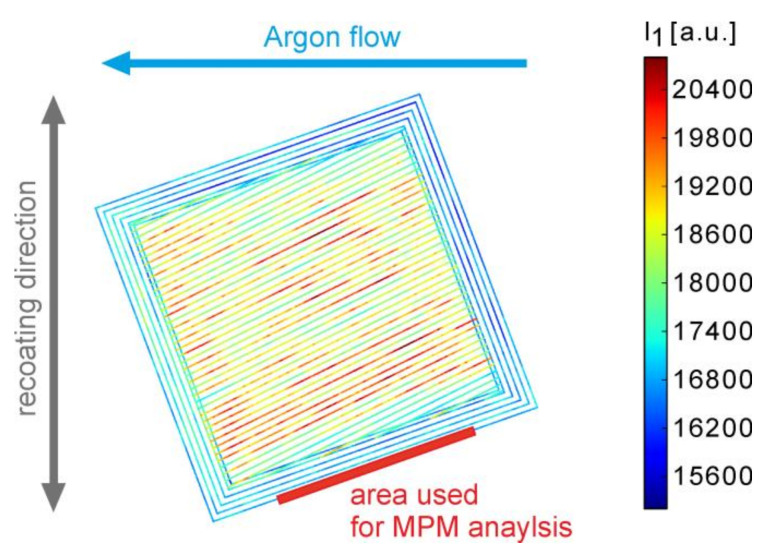
MPM (melt pool monitoring) photodiode intensity I_1_ for one layer of sample #05 (LPBF (laser powder bed fusion) parameters: 5 contour lines with a contour laser power P_cl_ = 100 W, contour velocity v_cl_ = 525 mm/s; volume laser power P = 175 W, velocity v = 500 mm/s, see Appendix A, Table A1). The data of the outer contour line within the red marked area was used for a consecutive comparison to roughness and stress results.

The SLM 280^HL^ machine used in this study is equipped with a commercial optical melt pool monitoring (MPM) system from SLM Solutions GmbH (Luebeck, Germany), consisting of two photodiodes in the near-infrared [46,47,48]. The emitted thermal radiation is acquired by both photodiodes and recorded in layer-wise fashion as values I_1_ and I_2_ dependent on the current laser scanner coordinates. An example of I_1_ for a typical cuboid sample layer is depicted in Figure 3.

The roughness and residual stress measurements were analyzed in the context of the MPM responses. Hence, the mean values and the standard deviations of the intensities I_1_ and I_2_, as well as the quotient I_2_/I_1_, were calculated from a similar region of each sample and along the building direction (marked in red in Figure 3). Both I_1_ and I_2_, as well as I_2_/I_1_, are presented below because I_1_ and I_2_ showed only qualitatively roughly similar trends, leading to a non-constant quotient. The data were filtered according to the following rules:

(i) only data points near the surface were considered from the selected region extending inwards 50 µm from the sample border;
(ii) only the middle part of the contour line was selected to exclude possible inhomogeneity towards the start or end of a specific scan vector. An offset of 1 mm towards the corners was chosen;
(iii) layers from a build height between 4 and 11 mm were evaluated;
(iv) for data reduction only, each fifth measurement value (i.e., one value each 50 µs) was considered.

Using these filtering rules, between 150 and 970 data points were obtained per layer depending on the chosen scanning velocity. In total, 233 layers were evaluated, meaning that at least 35,000 values were analyzed for each specimen.

### 2.3. Roughness Measurements

A confocal ZEISS LSM 700 laser scanning microscope (LSM), with an EC Epiplan-Apochromat 10x/0.3 HD DIC M27 (100× magnification) objective from Zeiss AG, Oberkochen, Germany, was used for roughness characterization. The same sample surface used for the MPM analysis (see Figure 3) and the residual strain measurements were characterized. A section in the middle of the cuboid’s side surface with a size of 1.75 × 1.75 mm^2^ was chosen with a lateral resolution of 1.25 µm (3 × 3 tiles with an overlap of 10%). Images from 90–100 different heights were taken for each sample, which corresponded to a resolution in the height of around 2.25–2.3 µm. The smallest available pinhole, a unidirectional scan direction, a digital gain of around 200, a medium laser power, and the second-highest scan speed were used. The measurement time amounted to around 30 min per specimen. Two different roughness properties—the arithmetic mean height S_a_ and the maximum height S_z_—were calculated according to DIN EN ISO 25178-2:2012-09 [49] using the ConfoMap 7 (ZEISS AG, Oberkochen, Germany) software. Representative LSM images are shown in Supplementary Figure S2.

### 2.4. Synchrotron X-ray Diffraction

Energy-dispersive diffraction experiments were conducted at the EDDI beamline of the synchrotron source BESSY II (Helmholtz Zentrum Berlin, Germany) [50]. The beamline exhibited a white beam (energy range 10–150 keV), which could be used to generate diffractograms dependent on the energy at a constant diffraction angle θ. The peak energy E_hkl_ (in keV) of the different crystallographic planes {hkl} was directly correlated to the corresponding lattice spacing d_hkl_:

(2) $$d_{\mathrm{hkl}}\left(Å\right)=\frac{6.199}{\sin\theta }\frac{1}{E_{\mathrm{hkl}}}$$

Several peak positions were obtained in each measurement that could be attributed to different penetration depths τ [51], depending on the linear absorption coefficient $\mu \left(E_{\mathrm{hkl}}\right)$ at the energy$\;E_{\mathrm{hkl}}$ [50]:

(3) $$\tau =\frac{\sin\theta }{2\mu \left(E_{hkl}\right)}\cos\psi$$

**Figure 4:**
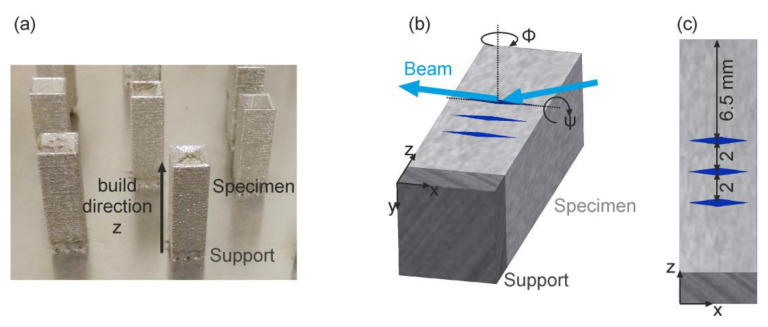
(**a**) Image of specimens on the base plate (specimen size 5 × 5 × 15 mm^3^ + 2 mm support); (**b**,**c**) simplified gauge volume shape and measurement points for measurement of strains in build direction z (ϕ = 0°). The identical sample sides were analyzed with MPM and diffraction (see Figure 1).

The chosen setup was identical to the one used in previous measurements [12,13,16]. All measurements were conducted in reflection at a diffraction angle of 2θ = 8°. With primary and secondary slit dimensions of 500 µm × 500 µm and 30 µm (vertical opening), respectively, a gauge volume length of 3.8 mm resulted (Figure 4; see further Ref. [20]). The residual stresses (RSs) were calculated with the *sin^2^ψ* method [52]. Importantly for the application of this method, (i) random crystallographic textures and (ii) negligible residual stresses (RSs) in the surface normal direction (σ_yy_ ≈ 0 MPa, i.e., a planar stress state) have to be assumed. Because experimentally obtained diffraction elastic constants were not available for as-built LPBF Ti-6Al-4V, diffraction elastic constants for α-Ti calculated according to the Eshelby–Kröner model and reported in [16] were used.

Residual stresses were investigated near the surface of small cuboids (5 × 5 × 15 mm^3^), as in previous studies [12,13]. Contrary to those studies, the surface plane (position y = 0) for the alignment of samples was defined by means of the integrated diffraction intensities instead of using a laser system. This was done because of the high roughness of the samples that can lead to erroneous positioning and, thus, erroneous determination of RS at the surface [13]. Integrated diffraction intensities near zero are obtained if the gauge volume is outside of the sample, whereas maximal intensities imply that the gauge volume is completely inside. Therefore, the y-position corresponding to half of the maximal intensity was defined as “surface” and set to be y = 0. This procedure was repeated for each measuring point and for each strain direction component (x and z).

In the previous studies, subsurface RSs were measured at 7 different points along one side of each sample (Figure 1a) [12,13]. Near the top of the specimens, stresses were low and increased towards the bottom until a stress plateau was reached at distances ≥ 6.5 mm. In order to use the measurement time effectively, only 3 positions within this stress plateau were measured (at 6.5 mm, 8.5 mm, and 10.5 mm from the top, see Figure 4c), and the average RS values were calculated in build direction σ_zz_ and perpendicular to it σ_xx_.

Further details regarding the choice of the {103}-reflection, the determination of the surface level, and the selection of the gauge depth for the RS are provided in the Supplementary.

Since only σ_zz_ was considered for analysis and discussion, the σ_xx_ values are only given in Supplementary Materials Table S1. Importantly, no correlation between σ_xx_ and σ_zz_ was evident (see Supplementary Figure S3).

The number of measured specimens was limited due to the available beamtime at the synchrotron facility. However, for future studies, the investigation of a larger number of specimens for further statistical analysis would be desired. This would enable characterizing for, e.g., the effects of control factors (see Ref. [53]).

## 3. Results

### 3.1. Effect of the Scan Pattern

Commonly, LPBF manufacturing is carried out with two different parameter sets—one set for the outer contour region, and one set for the volume of the part or specimen. However, in order to investigate the effect of the scan pattern, here, only *one* parameter set was used per sample (Table 1; for the sake of simplicity, all specimens are listed in Appendix A, Table A1). Specimens #01 and #02 were printed with only contour lines (CL) from the outside to the inside (O-I) and starting from the inside and finishing with the outside (I-O), respectively. Specimens #03 and #04 were printed only with volume hatching but using different laser parameters.

The particular laser parameters for the contour (#01, #02) were derived from the standard SLM Solutions contour strategy used for Ti-6Al-4V (i.e., for the contour: CL O-I). The parameters for the volume regions (#04) were based on the recent process window optimization presented in [54]. For sample #03, the contour parameters were adapted to the volume hatching for comparison.

P refers to the laser power, v to the scan velocity and h to the hatch distance used in the volume (V), or the contour region (CL), respectively. E_V_ corresponds to the volume energy density of the parameter set.

**Figure 5:**
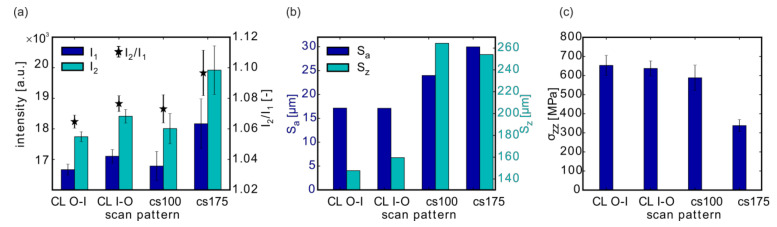
Influence of the scan pattern on the MPM data (**a**), on the surface roughness (**b**), and on the residual stresses (**c**). Depicted results are from samples #01 (CL O-I), #02 (CL I-O), #03 (chess scan strategy with P = 100 W), and #04 (chess/P = 175 W). CL O-I, contour lines from the outside to the inside; CL I-O, contour lines from the inside and finishing with the outside.

The MPM signals I_1_, I_2_, and I_2_/I_1_ were influenced mostly by the laser power P (Figure 5a): The samples produced with the same volume energy density E_v_ (#01–#03) showed lower MPM intensities than the sample with higher E_v_ (#04). The effects of changing scan strategies, in contrast, were much weaker in the MPM. When the scan order of the contour lines was inside to outside (i.e., #02; CL I-O), only slightly higher I_1_ and I_2_ responses were obtained compared to the reverse sequence. In the case of printing the outer contour line last (CL I-O), the inner volume had already been molten and would still be hot. As a result, the heat loss caused by heat conduction to the surrounding material would be smaller for the last contour line, possibly leading to slightly higher melt pool temperatures and thermal emissions. The photodiodes would not only acquire photons emitted from the melt pool but also from the hot surrounding material. Both factors would contribute to the higher MPM intensities for #02 compared to #01.

For samples #03 and #4, produced only with volume hatching using the chess pattern and different laser powers, mainly the high standard deviations in the MPM data stood out. This was likely caused by scan lines that start or end in the detection area since usually more unstable and varying melt conditions prevail at the terminal points of the scan vectors.

For the surface roughness (Figure 5b), the role of the scan patterns was more important than E_V_. Smaller surface roughness is generally obtained when contour lines are used [6]. In this case, the use of contour lines reduced the arithmetic mean roughness S_a_ by a factor of ~2.

The influence of the scan pattern on the residual stresses (Figure 5c) depended on the stress component. For the residual stresses σ_zz_ in build direction, E_V_ dominated the effects: higher σ_zz_ values were measured for samples produced at lower volume energy densities (#01–#03). This was consistent with previous studies [12,13,16].

### 3.2. Effect of the Scan Order

Samples #05 to #08 were printed in order to analyze the role of the scan order of the volume (V) and contour lines (CL). The volume process parameters were fixed at P = 175 W, v = 500 mm/s, and h = 100 µm. The contour consisted of five contour lines and one fill contour line, all of which were printed with P_CL_ = 100 W, v_CL_ = 525 mm/s, and h_CL_ = 90 µm.

The following samples with their particular scan sequences were investigated (Table 2):

P refers to the laser power, v to the scan velocity and h to the hatch distance used in the volume (V), or the contour region (CL), respectively. E_V_ corresponds to the volume energy density of the parameter set.

**Figure 6:**
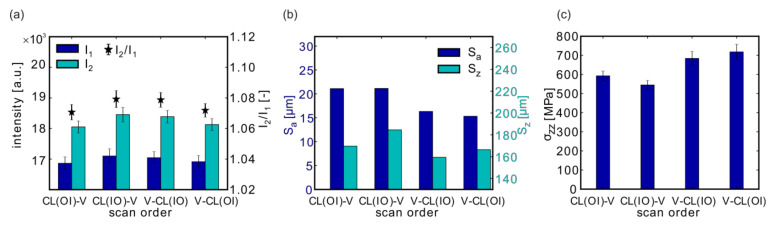
Influence of the scan order on the MPM data (**a**), on the surface roughness (**b**), and on the residual stresses (**c**). Depicted are the results from samples #05 (CL(O-I)-V), #06 (CL(I-O)-V), #07 (V-CL(I-O)), and #08 (V-CL(O-I)).

Again, the recorded MPM intensities I_1_ and I_2_ were slightly higher in case of scanning the contour lines from the inside to the outside (Figure 6a; samples #06 (CL (I-O)-V) and #07 (V-CL (I-O)).

The surface roughness S_a_ and S_z_ (Figure 6b) was slightly reduced when the inner volume was scanned before the outer contour vectors. In accordance with the results from the scan pattern variations #01 (CL (O-I) and #02: CL (I-O)), the scan order of the contour lines seemed to be less relevant for the studied coupons.

A contrary effect was observed for the residual stresses in the build direction (Figure 6c): σ_zz_ was slightly higher when either the volume was scanned first or when the scan order of the contour lines was “outside to inside”.

### 3.3. Effect of the Number of Contour Lines

In the standard SLM Solutions scan strategy for Ti-6Al-4V, two contour lines were used (i.e., CL O-I, followed by volume hatching, #09) (Standard SLM Solutions scan strategy for Titanium in Metal Build Processor 2.1, setting Ti_SLM_BP2.1_30_Stripes-US_T200_S21-01_V5002). In order to investigate their influence, the contour vectors were increased from 2 (sample #09) to 5 (#05) and 10 (#10). The same volume process parameters (P = 175 W; v = 500 mm/s; h = 100 µm) and the same contour parameters (P_CL_ = 100 W; v_CL_ = 525 mm/s; h_CL_ = 90 µm) were chosen (Table 3). The scan order for the contour lines was “outside to inside” (before volume) for all specimens. These specimens could be compared to those manufactured either without contour lines (only volume hatching; #04) or the one printed merely with contour vectors (#01).

**Figure 7:**
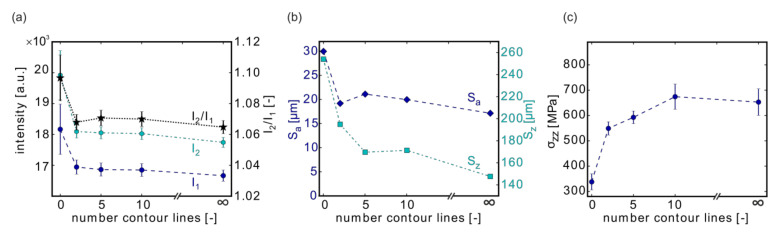
The influence of the contour line count on the MPM data (**a**), on the surface roughness (**b**), and on the residual stresses (**c**). Depicted are the results from samples #04 (only volume, no contour lines), #09 (2 contour lines), #05 (5 contour lines), #10 (10 contour lines), and #01 (only contour lines, no volume). Note: The lines connecting the measurement points are only provided as a guide for the eye.

The MPM signals, as well as the roughness, showed similar trends with the highest values obtained in the case of pure volume hatching (Figure 7a). Consistent with the observations in Section 3.1, the higher E_v_ of the volume hatching versus the contour parameters was mainly responsible for the higher MPM intensities when no contour vectors were used.

The higher roughness was related to the scan pattern, i.e., numerous scan vectors ending or starting on the sample edge causing higher S_a_ values than scan vectors paralleling the sample edge (Figure 7b). Thus, when contour lines were used, the MPM intensities and the roughness decreased substantially. The chosen number of contour lines appeared to be secondary. Because the outermost contour line was scanned first, the rest of the scan pattern on the inside became less relevant, at least for MPM and the surface quality.

However, the number of contour lines strongly affected the residual stresses (Figure 7c). The σ_zz_ component seemed to converge on a plateau value for more than 10 contour lines. Most of the increase of the residual stress component σ_zz_ was obtained with the first five contour lines used.

### 3.4. Variation of Main Laser Parameters

The contour laser power P_CL_, scan velocity v_CL_, and the hatch distance h_CL_ were varied in the next step (Table 4). The number of contour lines and the volume parameters were kept constant.

#### 3.4.1. Effect of Laser Power

**Figure 8:**
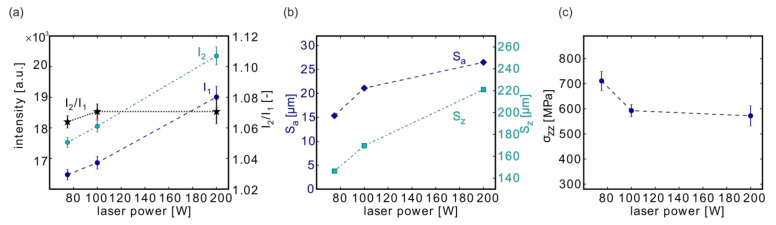
Influence of the power on the MPM data (**a**), on the surface roughness (**b**), and on the residual stresses (**c**). Depicted are the results from samples #11 (P_CL_ = 75 W), #05 (P_CL_ = 100 W), #12 (P_CL_ = 200 W). Note: The lines connecting the measurement points are only provided as a guide for the eye.

The influence of the laser power was studied with the samples manufactured with three different contour laser powers P_CL_ = 75 W (#11), 100 W (#05), 200 W (#12) (Figure 8).

The MPM intensities increased with P_CL_ (Figure 8a), which is in agreement with simulations from Ref. [55] that show a rise in melt pool temperature with higher laser power. Due to the different sensitivity ranges of both photodiodes of the SLM Solution melt pool monitoring system and the shift in the wavelength of the thermal radiation maximum, the intensities rose in a different manner, and the intensity quotient did not change significantly. Only the scatter increased with higher laser power P.

The roughness showed a clear tendency in line with the results from the previous sections to increase at higher laser powers (Figure 8b). Conversely, σ_zz_ yielded lower stresses when the energy density rose (Figure 8c). However, because only three different laser powers were investigated, the exact behavior between 100 W and 200 W was not well resolved.

#### 3.4.2. Effect of the Scanning Velocity

The influence of the scanning velocity was investigated with samples #13 (contour scanning velocity v_CL_ = 250 mm/s), #05 (v_CL_ = 525 mm/s), and #14 (v_CL_ = 1050 mm/s).

**Figure 9:**
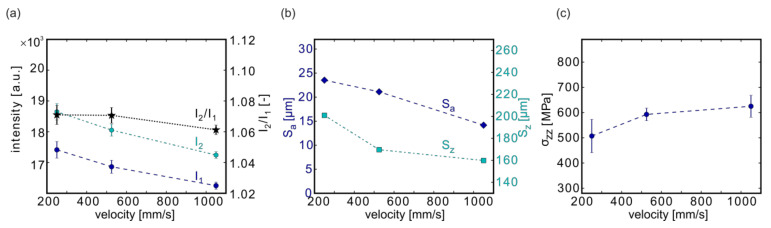
Influence of the scanning velocity on the MPM data (**a**), on the surface roughness (**b**), and on the residual stresses (**c**). Depicted are the results from samples #13 (250 mm/s), #05 (525 mm/s), and #14 (1050 mm/s). Note: The lines connecting the measurement points are only provided as a guide for the eye.

Both MPM intensities and the intensity quotient decreased with increasing contour scanning velocity (Figure 9a). This is qualitatively in accordance with higher melt pool temperatures obtained in simulations with decreasing scan velocities [56]. The same decreasing trend was observed for the roughness (Figure 9b; S_a_: From 23.5 to 14.2 µm), whereas the stresses σ_zz_ in build direction (Figure 9c) showed an increase from 507 to 625 MPa.

#### 3.4.3. Effect of the Hatch Distance

For investigating the influence of the hatch distance on surface quality and residual stresses, samples with three different contour hatch distances were used: #15 (h_CL_ = 60 µm), #05 (h_CL_ = 90 µm), and #16 (h_CL_ = 120 µm).

**Figure 10:**
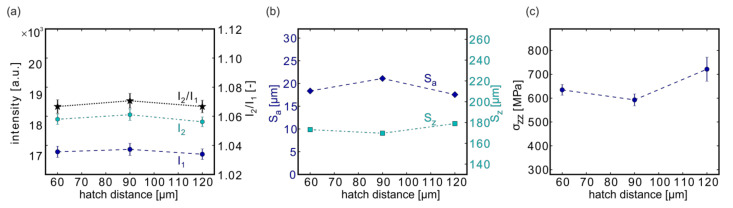
Influence of the hatch distance on the MPM data (**a**), on the surface roughness (**b**), and on the RS (**c**). Depicted are the results from samples #15 (60 µm), #05 (90 µm), and #16 (120 µm). Note: The lines connecting the measurement points are only provided as a guide for the eye.

Only a slight influence of the hatch distance on the MPM data and the surface roughness could be observed in the investigated parameter range (Figure 10a,b). However, the typical variations caused by the LPBF process were of a similar order of magnitude. The influence on the residual stresses was slightly more pronounced, and higher stresses were obtained for larger hatch distances (Figure 10c).

### 3.5. Effect of Changing P, v, and h at E_v_ = const.

In the experiments described in Section 3.4, each laser parameter was varied separately. In the following set of samples, several laser parameters were changed simultaneously such that E_v_ was kept constant. Again, only a contour scan strategy was used and, thus, P_CL_, v_CL_, and h_CL_ were varied (Table 5).

**Figure 11:**
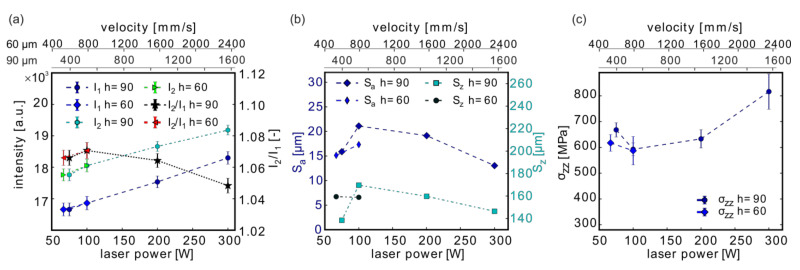
Effects of the laser power, scanning velocity, and hatch distance at constant volume energy density (E_v_ = const.) on the MPM data (**a**), on the surface roughness (**b**), and on the residual stresses (**c**). Depicted are the results of the samples listed in Table 5. The upper-velocity axis at the top of each Figure corresponds to the h = 60 µm and the lower one to the h = 90 µm curves. Note: The lines connecting the measurement points are only provided as a guide for the eye.

The MPM intensities generally increased with the linked increase in laser power and scanning velocity (Figure 11a). If both laser parameters acted similarly, the MPM intensity should remain constant, such as E_V_. However, the increase indicated that the influence of the laser power on the MPM intensity dominated the influence of the scanning velocity.

Comparable to the results of the independent power variation (Figure 8a), the trend of the intensity quotient I_2_/I_1_ differed from that of the single photodiode intensities. A maximum of I_2_/I_1_ was reached at P_CL_ = 100 W, which decreased strongly towards 300 W (Figure 11a). The different hatch distances, in contrast, did not influence the MPM response.

The roughness S_a_ (Figure 11b) followed the trend of the MPM intensity quotient. The surface roughness was rather low for a low laser power (and low v) as well as for the highest chosen laser power (and v). At P_CL_ = 300 W and 1575 mm/s, the lowest surface roughness S_a_ ≈ 13 µm, in this study, was achieved.

The residual stresses opposed the roughness trend: they were higher for a low laser power (low v) as well as for a high laser power (high v), while in between, a minimum in the stresses was observed. In the independent single parameter variations (Section 3.4), σ_zz_ increased with decreasing laser power and increased with higher scanning velocities. The observed trend with a simultaneous variation of P and v at E_V_ = const. suggests a change of the key factors when viewed in the context of the single parameter variations for P (Section 3.5; Figure 8c) and v (Figure 9c). For the combination of low P and low v, the laser power appeared to be the important factor, leading to the slight σ_zz_ increase (a strong increase was observed for decreasing P, Figure 8c), whereas, at high P and high v, the scanning velocity might dominate the residual stress increase (increase at larger v, Figure 9c).

### 3.6. Effect of a Post-Processing Surface Treatment–Shot Peening

**Figure 12:**
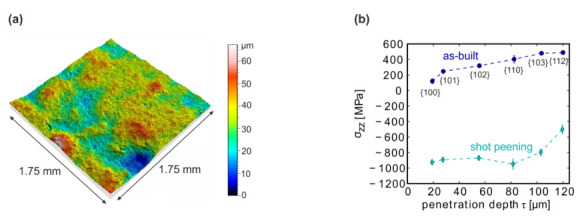
(**a**) LSM topography results of the shot-peened specimen (#22); (**b**) stress depth profile for a shot-peened specimen relative to an as-built specimen (#09 for y = 0 µm). Each stress value belongs to a certain {hkl}-peak, which is given in the brackets. The penetration depth τ was calculated according to Equation (3).

One specimen (#22), manufactured with the same LPBF processing parameters as used for sample #09, was shot-peened in a post-processing step. As a result, the surface roughness was reduced to S_a_ = 6.3 µm and S_z_ = 67.5 µm (Figure 12a). Furthermore, high compressive residual stresses in a range of −800 ± 40 MPa were now obtained (from the {103}-Ti reflection at around 100 µm). The stresses were measured at the surface (y = 0 µm). The compressive stresses were comparable to those of ca. −910 MPa reported for a similar treatment by Leuders et al. (R_a_ therein 4.2 µm after shot peening) [4,8].

The high compressive stresses in coupon #22 prevailed at the surface and were almost constant up to a penetration depth of 80 µm. In comparison, almost no stresses could be measured at the surface (y = 0 µm) of the as-built specimen, and only farther inside than ~100 µm tensile stresses were detected (see Methods Section for the choice of the sampling depth).

## 4. Discussion

### 4.1. Identification of the Dominating Influence Factors from Laser Power, Scan Velocity, and Hatch Distance on MPM, Surface Quality, and Residual Stresses

**Figure 13:**
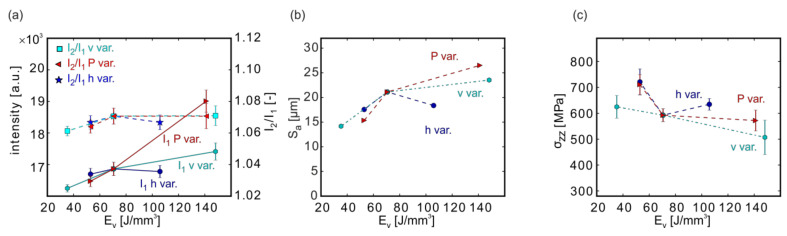
Comparison of the results for the variation of the hatch distance h, laser power P, and scanning velocity v in relation to E_V_ (details see Section 3.4) on the MPM data (**a**), on the surface roughness (**b**), and on the residual stresses (**c**).

The influence of the contour laser parameters (power, scanning velocity, and hatch distance) is presented separately in the Results Section 3.4. Rather than discussing the separate influences, the main observations shall be analyzed on a deeper level. The energy density E_V_ is used in place of the specific laser process parameters (Figure 13) to identify trends that can assist LPBF parameter developments.

The MPM intensities I_1_ and I_2_ (I_1_ is given in Figure 13a, I_2_ is omitted for clarity) showed a positive correlation with E_V_, which was due to raising the laser power P or slowing the scanning velocity v. The laser power had the largest influence on the intensity. This was in accordance with recent simulations that concluded that variations of the laser power had a larger influence on the melt pool temperature (and local temperature gradient) than variations of the scanning velocity [55,56]. The variation of the hatch distance h only had a small influence on the MPM intensities of the outermost contour line because the inner contour and fill contour lines were printed after the outermost contour vector.

In contrast to the MPM single diode intensities, the quotient I_2_/I_1_ provided a slightly different behavior (Figure 13a). For low E_V_, the quotient was smaller, but the strong increases in the single intensities canceled out, and I_2_/I_1_ stayed almost constant towards higher E_V_. For the scanning velocity and the laser power variation, a plateau of the quotient around I_2_/I_1_ ≈ 1.07 was reached for E_V_ ≥ 70 J/mm^3^.

Analogous to the single MPM intensities I_1_ and I_2_, the laser power had the highest influence on the roughness with the higher S_a_ values being obtained for increasing P. The residual stresses showed an opposite trend: stresses in build direction σ_zz_ tended to decrease with increasing E_v_, which was in accordance with previous results [16]. However, relative to the comparably large variations of S_a_ (~14.2 to 26.5 µm) with E_V_, the bandwidth of σ_zz_ was smaller (~500–720 MPa). Hence, S_a_ appeared to be more sensitive to E_V_ than σ_zz_ within this set of samples.

### 4.2. Correlation between Surface Roughness and Residual Stresses

All measured residual stresses of the as-built specimens were tensile in nature, independent of the chosen scan pattern, scan order, or the chosen process parameters. The stresses in build direction σ_zz_ (between 338 MPa and 816 MPa) were affected particularly strongly by the laser power, but also by the scan velocity (Figure 13a). The lowest σ_zz_ = 338 MPa was obtained for sample #04, which was produced only with volume hatching at comparably high P = 175 W, resulting in a high E_v_. In order to achieve overall low stresses σ_zz_, low velocities (Figure 9c) and the scan order “contour first (inside-outside), then volume hatching” (Figure 6) were favorable when contour and volume parameters were used together.

The arithmetic mean heights S_a_ obtained in this study for the group of as-built samples varied between 13 µm and 30 µm and maximum heights S_z_ between 138 µm and 264 µm, suggesting that the use of an optimized LPBF contour strategy, as already generally recommended by machine producers, could improve surface qualities by a factor of up to ~2. High velocities, in combination with high laser powers, seem to be most promising in terms of a low roughness. The lowest values of S_a_ reported in this study based on confocal microscopy were in a good agreement with those reported from characterization with contact-based 1D methods for LPBF Ti-6Al-4V: Greitemeier et al. [7] obtained roughness values of R_a_ ≈ 13.0 µm, and Leuders and coworkers [8] reported a value of ca. 18 µm for the corresponding surface orientation (90°), with slightly different specimen geometries as used in this study.

**Figure 14:**
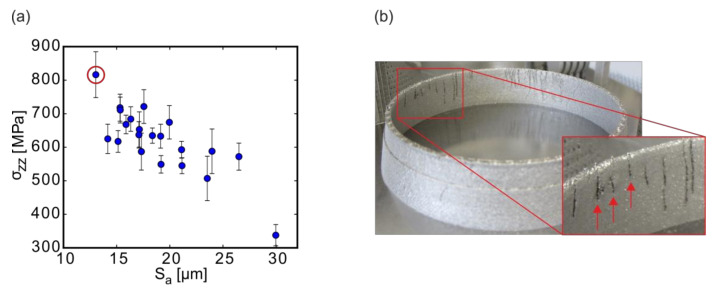
Correlation of the mean arithmetic height S_a_ with (**a**) the stress in build direction σ_zz_ and (**b**) image of a component (diameter ~80 mm, height ~15 mm) built with the same parameters that were used for sample #19 and exhibited severe RS-induced cracking. Three examples of cracks are indicated by red arrows.

Importantly, the optimization of the contour properties has to be viewed in the context of the correlation with the residual stresses because both S_a_ and S_z_ were linked to σ_zz_ (Figure 14). High roughness values were correlated to low residual stresses and vice versa (Figure 14a).

This reminds of Pandora’s box: the roughness cannot be reduced without negatively affecting another important property, the residual stress. In fact, when using the contour strategy, giving the lowest, i.e., best roughness results (i.e., sample #19), the residual stresses in build direction exceeded tolerable limits and led to substantial cracking in manufactured components. This can be seen in the example of a fuel injector ring with fine channels in Figure 14b (The stl.-file of the injector element was created with the CAD-software Inventor). While the surface roughness was low, the residual stresses were so large that cracks developed in the contour region near the internal channels during the build.

This example highlights that in AM process developments, each optimization step has to be carried out carefully, taking into account correlations, because typically different properties are affected differently at the same time. One explanation for the correlation of σ_zz_ and S_a_ in the case of high tensile stresses might be that the melt may have a high tendency to shrink (due to surface tension as evidenced, e.g., by balling effects) during the cooling process. If the melt tends to shrink, on the one hand, fewer powder particles will be attached to the welding tracks during solidification, lowering the surface roughness in turn. On the other hand, however, a high tendency to shrink could explain the simultaneously high residual stresses observed for the respective scan strategies, exhibiting improved surface qualities.

### 4.3. Correlation of MPM to Surface Roughness and Residual Stresses

**Figure 15:**
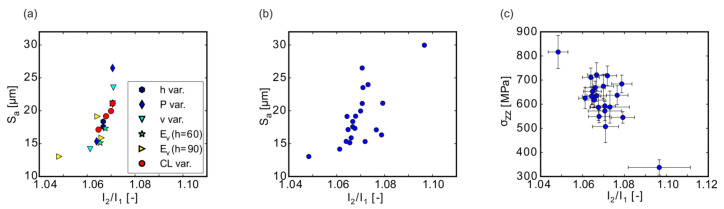
(**a**) Correlation of S_a_ with I_2_/I_1_ for specimens, which were manufactured with contour lines and the standard scan order CL(O-I)-V. The following samples are depicted: hatch distance variation (h var.), Section 3.4. (**c**); laser power variation (P. var), Section 3.4. (**a**); velocity variation (v var.), Section 3.4. (**b**); samples from the studies with hatch distances of 60 µm and = 90 µm at E_V_ = const. (h = 60 µm and h = 90 µm), Section 3.5; samples from the contour line (CL var), Section 3.3. (**b**) Correlation of S_a_ with I_2_/I_1_ for all specimens. (**c**) Correlation of the residual stress in build direction σ_zz_ with I_2_/I_1_.

An analysis of the correlation between the MPM data and the arithmetic mean surface height S_a_ showed that the monitoring might be used for obtaining a first prediction of the surface roughness that would be obtained with a specific LPBF parameter set (Figure 15a). The correlation was found to be better at the I_2_/I_1_ quotient than to any of the single intensities (see Supplementary Figure S4 for correlations to I_1_). The correlation obtained for similar scan orders in Figure 15a related to higher MPM I_2_/I_1_ values to higher surface roughness. For the specimens manufactured with high laser power belonging to the P variation (Section 3.4), or E_v_ = const (h = 90 µm) or E_v_ = const. (h = 60 µm) series (Section 3.5) in Figure 15a, the trend was maintained, but it is striking that the samples belonging to the variation of the contour line number (CL var. in Figure 15a; Section 3.3) showed the best correlation. This might indicate that—once the correlation between roughness and MPM is known for one determined parameter set—the roughness can be predicted from the MPM data, which would be a considerable step towards quality monitoring.

Importantly, this correlation works best in cases following the same scan strategy, i.e., particularly the scan order (cp. Figure 15a,b): While (a) contains only data points following a similar scan order (standard: contour lines (outside to inside), followed by volume) and provides a good correlation between I_2_/I_1_ and S_a_, (b) also contains additional data points from the samples manufactured with different scan orders. A better correlation for similar scan orders can be understood from the behavior observed in Section 3.2: the MPM intensities were influenced sizably by the scan order of the contour lines (I-O versus O-I; Figure 6a), but the surface roughness was affected only slightly (Figure 6b). Hence, the estimate of the roughness value from the MPM data will be less reliable if the scan order in the contour region is changed.

Similarly, the MPM signals, again particularly I_2_/I_1_, and the residual stresses of the respective samples seemed to follow a rough correlation that might provide a means to estimate residual stresses from in situ process monitoring (Figure 15). This is not surprising in the light of the correlation between S_a_ and σ_zz_ discussed above (Figure 14): samples having shown high MPM intensities during LPBF consequently exhibited lower stresses in build direction σ_zz_.

## 5. Conclusions

In this study, 21 different scan strategies in LPBF of Ti-6Al-4V were studied systematically at the coupon level, and their effects on surface qualities and subsurface residual stresses were analyzed. Correlations between the laser scan strategies, the respective laser parameters, and the surface roughness, as well as the residual stresses (in the as-built condition), were identified. The LPBF build was monitored in situ with the integrated photodiode-based melt pool monitoring system. The following main results were obtained:

- S_a_ and near-surface residual stresses σ_zz_ were intrinsically linked in the LPBF specimens.
- The ranges obtained for S_a_ and σ_zz_ implied a large potential for an optimization of the LPBF parameters, although due to the link between both and their inverse dependence on the laser power and scan velocity, this optimization might only result in a Pareto optimum. For the investigated sample geometry, the stresses in the build direction σ_zz_ increased up to a level at which macroscopic cracking in manufactured components occurred, when contour strategies focusing primarily on lowering the surface roughness were employed.
- Therefore, any parameter optimization has to be carried out carefully. An optimal contour scan strategy with vectors processed first from the outside and then on the inside (at 100 W, 1050 mm/s, and 90 µm contour hatch distance) prior to volume hatching can be recommended based on the analyzed Ti-6Al-4V samples. This strategy represents a trade-off between RS and roughness (σ_zz_ = 625 MPa, S_a_ = 14.2 µm).
- The MPM monitoring may be suitable to estimate the surface roughness from the intensity quotient I_2_/I_1_ of the two photodiodes: A correlation between I_2_/I_1_ and S_a_ was discovered particularly for groups of samples manufactured with similar contour strategies, e.g., for samples printed with the scan order “contour vectors (from outside to the inside), followed by volume hatching”. Due to the link between S_a_ and σ_zz_, the MPM data I_2_/I_1_ can also be used to estimate the resulting residual stresses in the LPBF material. The predictive estimates of S_a_ and σ_zz_ from the melt pool monitoring during the LPBF process can be useful for the development of build strategies and parameter adaption in complex components.

Further studies are required in order to elucidate and transfer these results to different coupon geometries exhibiting, e.g., up- and down-skin surfaces. The current results may provide helpful insights for the analysis of the underlying mechanisms for residual stress build-up and redistribution from thermal simulations of the LPBF process.

## Figures and Tables

**Table 1:** Samples built with a single scan strategy of different scan patterns consisting either of contour or volume scan vectors.

No	Scan Order	Scan Strategy Contour	Scan Strategy Volume	Contour Parameters				Volume Parameters			
				Power P_CL_ (W)	Scan vel. v_CL_ (mm/s)	Hatch h_CL_ (µm)	E_v,cl_ (J/mm^3^)	Power P (W)	Scan vel. v (mm/s)	Hatch H (µm)	E_v_ (J/mm^3^)
#01	-	CL O-I	-	100	525	90	71	-	-	-	-
#02	-	CL I-O	-	100	525	90	71	-	-	-	-
#03	-	-	Chess (cs100)	-	-	-	-	100	525	90	71
#04	-	-	Chess (cs175)	-	-	-	-	175	500	100	117

**Table 2:** Samples build with different scan orders.

No	Scan Order	Scan Strategy Contour/# of Contour Lines	Scan Strategy Volume	Contour Parameters				Volume Parameters			
				Power P_CL_ (W)	Scan vel. v_CL_ (mm/s)	Hatch h_CL_ (µm)	E_v,cl_ (J/mm^3^)	Power P (W)	Scan vel. v (mm/s)	Hatch h (µm)	E_v_ (J/mm^3^)
#05	CL-V	CL (O-I) / 5	Chess	100	525	90	71	175	500	100	117
#06	CL-V										
#07	V-CL										
#08	V-CL										

**Table 3:** Samples used to compare different contour line counts.

No	Scan Order	Scan Strategy Contour/# of Contour Lines	Scan Strategy Volume	Contour Parameters				Volume Parameters			
				Power P_CL_ (W)	Scan vel. v_CL_ (mm/s)	Hatch h_CL_ (µm)	E_v,cl_ (J/mm^3^)	Power P (W)	Scan vel. v (mm/s)	Hatch h (µm)	E_v_ (J/mm^3^)
#04	V	- 0	Chess	-	-	-	-	175	500	100	
#09	CL-V	CL (O-I) 2	Chess	100	525	90	71	175	500	100	117
#05	CL-V	CL (O-I) 5	Chess	100	525	90	71	175	500	100	117
#10	CL-V	CL (O-I) 10	Chess	100	525	90	71	175	500	100	117
#01	CL	CL (O-I) ∞	-	100	525	90	71	-	-	-	-

**Table 4:** Varying the main laser parameters, E_V_ = variable.

No	Scan Order	Scan Strategy Contour/# of Contour Lines	Scan Strategy Volume	Contour Parameters				Volume Parameters			
				Power P_CL_ (W)	Scan vel. v_CL_ (mm/s)	Hatch h_CL_ (µm)	E_v,cl_ (J/mm^3^)	Power P (W)	Scan vel. v (mm/s)	Hatch H (µm)	E_v_ (J/mm^3^)
#05	CL-V	CL (O-I) / 5	Chess	100	525	90	71	175	500	100	117
#11				75	525	90	53				
#12				200	525	90	141				
#13				100	250	90	148				
#14				100	1050	90	35				
#15				100	525	60	106				
#16				100	525	120	53				

**Table 5:** Varying the main laser parameters, E_V_ = const.

No	Scan Order	Scan strategy Contour/# of Contour Lines	Scan Strategy Volume	Contour Parameters				Volume Parameters			
				Power P_CL_ (W)	Scan vel. v_CL_ (mm/s)	Hatch h_CL_ (µm)	E_v,cl_ (J/mm^3^)	Power P (W)	Scan vel. v (mm/s)	Hatch H (µm)	E_v_ (J/mm^3^)
#05	CL-V	CL(O-I)/5	Chess	100	525	90	71	175	500	100	117
#17				75	394	90	71				
#18				200	1050	90	71				
#19				300	1575	90	71				
#20				66.7	525	60	71				
#21				100	787.5	60	71				

## Supplementary Materials

The following materials are available online at https://www.mdpi.com/1996-1944/13/15/3348/s1, Table S1: Measured values: MPM data, surface roughness data, and residual stress components, Figure S1: Diffractograms for different ψ-angles, measuring depths, integrated peak intensities and RS in build direction, Figure S2: LSM topology data of selected samples, Figure S3: The analysis of the potential correlations between stresses perpendicular to the build direction σ_xx_ and in-build direction σ_zz_, Figure S4: Comparison of S_a_ with I_1_ as well as σ_zz_ with I_1_ for all specimens.

## Appendix A

**Table A1:** Summary of all specimens manufactured with LPBF using different scan strategies for the current study. Sample #09 corresponds to the SLM Solution standard scan strategy.

No	Scan Order	Scan Strategy Contour/# of Contour Lines	Scan Strategy Volume	Contour Parameters				Volume Parameters				Scan Pattern
				Power P_CL_ (W)	Scan vel. v_CL_ (mm/s)	Hatch h_CL_ (µm)	E_v,cl_ (J/mm^3^)	Power P (W)	Scan vel. v (mm/s)	Hatch h (µm)	E_v_ (J/mm^3^)	
#01	CL	CL(O-I) /∞	-	100	525	90	71	-	-	-	-	
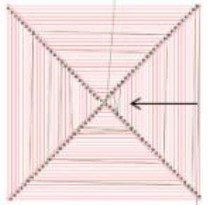
#02	-	CL(I-O)/∞	-	100	525	90	71	-	-	-	-	
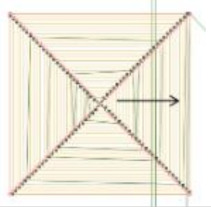
#03	V	-/0	Chess	-	-	-	-	100	525	90	71	
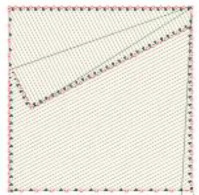
#04	V	-/0	Chess	-	-	-	-	175	500	100	117	
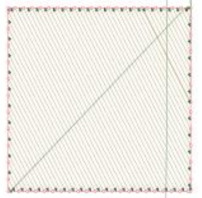
#05	CL-V	CL(O-I)/5	Chess	100	525	90	71	175	500	100	117	
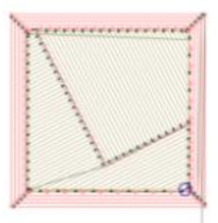
#06	CL-V	CL(I-O)/5	Chess	100	525	90	71	175	500	100	117	
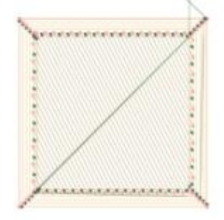
#07	V-CL	CL(I-O)/5	Chess	100	525	90	71	175	500	100	117	
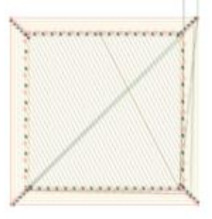
#08	V-CL	CL(O-I)/5	Chess	100	525	90	71	175	500	100	117	
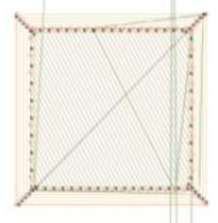
#09	CL-V	CL(O-I)/2	Chess	100	525	90	71	175	500	100	117	
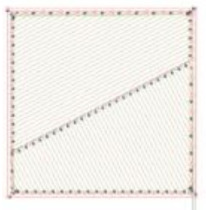
#10	CL-V	CL(O-I)/10	Chess	100	525	90	71	175	500	100	117	
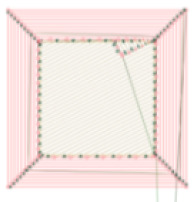
#11	CL-V	CL(O-I)/5	Chess	75	525	90	53	175	500	100	117	
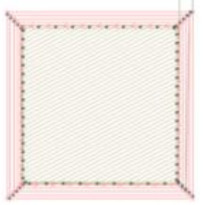
#12	CL-V	CL(O-I)/5	Chess	200	525	90	141	175	500	100	117	
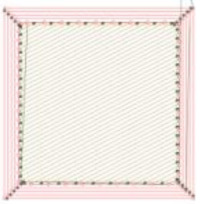
#13	CL-V	CL(O-I)/5	Chess	100	250	90	148	175	500	100	117	
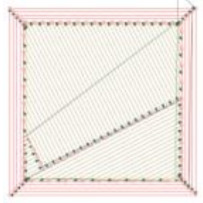
#14	CL-V	CL(O-I)/5	Chess	100	1050	90	35	175	500	100	117	
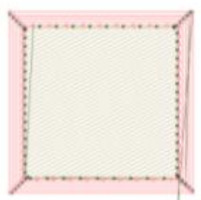
#15	CL-V	CL(O-I)/5	Chess	100	525	60	106	175	500	100	117	
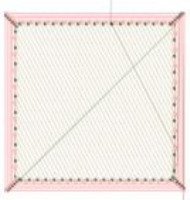
#16	CL-V	CL(O-I)/5	Chess	100	525	120	53	175	500	100	117	
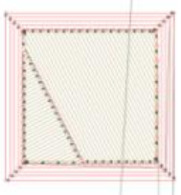
#17	CL-V	CL(O-I)/5	Chess	75	394	90	71	175	500	100	117	
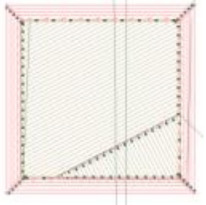
#18	CL-V	CL(O-I)/5	Chess	200	1050	90	71	175	500	100	117	
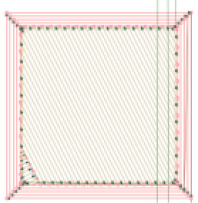
#19	CL-V	CL(O-I)/5	Chess	300	1575	90	71	175	500	100	117	
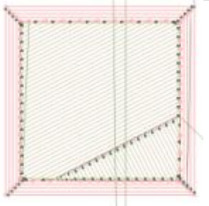
#20	CL-V	CL(O-I)/5	Chess	66.7	525	60	71	175	500	100	117	
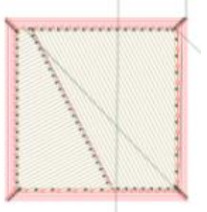
#21	CL-V	CL(O-I)/5	Chess	100	787.5	60	71	175	500	100	117	
