# Supplementary material for: Pandora’s Box–Influence of Contour Parameters on Roughness and Subsurface Residual Stresses in Laser Powder Bed Fusion of Ti-6Al-4V
Source: Materials (Basel). 2020 Jul 28;13(15):3348. doi: 10.3390/ma13153348 (PMC7436019; doi:10.3390/ma13153348)
Supplement: Supplementary file 1 [file materials-13-03348-s001.pdf]

materials

MDPI

Article

Pandora's Box–Influence of Contour Parameters on Roughness and Subsurface Residual Stresses in Laser Powder Bed Fusion of Ti-6Al-4V

Supplementary Material

Katia Artzt [1,*], Tatiana Mishurova [2], Peter-Philipp Bauer [1,] Joachim Gussone [1], Pere Barriobero-Vila [1], Sergei Evsevleev [2], Giovanni Bruno [2,3], Guillermo Requena [1,4] and Jan Haubrich [1]

1. Institute of Materials Research, German Aerospace Center (DLR; Deutsches Zentrum für Luft- und Raumfahrt), Linder Höhe, 51147 Cologne, Germany; Peter-Philipp.Bauer@dlr.de (P.-P.B.); joachim.gussone@dlr.de (J.G.); pere.barrioberovila@dlr.de (P.B.-V.); guillermo.requena@dlr.de (G.R.); jan.haubrich@dlr.de (J.H.)
2. Bundesanstalt für Materialforschung und–prüfung (BAM; Federal Institute for Materials Research and Testing), Unter den Eichen 87, 12205 Berlin, Germany; tatiana.mishurova@bam.de (T.M.); sergei.evsevleev@bam.de (S.E.); giovanni.bruno@bam.de (G.B.)
3. Institute of Physics and Astronomy, University of Potsdam, Karl-Liebknecht-Straße 24/25, Potsdam 14476, Germany
4. Metallic Structures and Materials Systems for Aerospace Engineering, RWTH Aachen University, 52062 Aachen, Germany

* Correspondence: katia.artzt@dlr.de

Received: 29 June 2020; Accepted: 24 July 2020; Published: date

Further experimental details on the residual stress measurements: Selection of the {103}-reflection and the gauge depth

Diffractograms for 8 different ψ-angles (example see Figure S1 (a)) were measured in two ϕ-directions (the position of gauge volume is shown in Figure 4 (c, d)). The diffraction patterns comprise peaks of 8 crystallographic planes of the (α / α') hexagonal Ti lattices (**Error! Reference source not found.** (a)). The lowest intergranular stresses are reached for the {103}-peak [1, 2]. As a result the {103}-reflection was predominantly used in this study to calculate residual stresses.

Typically, some residual powder particles remain attached at the surface position of the LPBF material after the process. Consequently, only low macrostresses prevail at y = 0, which are not representative for the specimen's manufacturing parameters. Therefore, the subsurface RS measurements were conducted at another position shifted into the volume (y > 0): at first, diffraction patterns at different y-positions were acquired (Figure S1 (b)) and the intensities (Figure S1 (c)) as well as the stresses (Figure S1 (d)) for the α-Ti {103} reflection evaluated. Peak intensities and RS increase towards the bulk and the intensity reaches a maximum at around y = 150 μm for ψ = 0°; the stress is highest for about y ≈ 200 μm.

It is striking that the diffractograms and the intensities change for y > 150 μm (Figure S1 (b), (c)), which indicates that the material is more textured in the bulk for y > 150 μm (see e.g. **Error! Reference source not found.** (c), 300 μm data set) consistent with previous reports on bulk coupons [3, 4]. Therefore, to avoid violating the assumptions of texture isotropy and a plane stress state for the analysis, we decided to choose y = 150 μm as measuring position, which also coincided with the best detected diffraction intensities. One exception was made for two specimens, which were printed without contour lines (specimens #03 (volume parameters P = 100 W, v = 525 mm/s) and #04 (P = 175 W, v = 500 mm/s)) and thus exhibit a much higher surface roughness. A measurement depth of y = 250 μm was chosen in these cases instead of y = 150 μm.

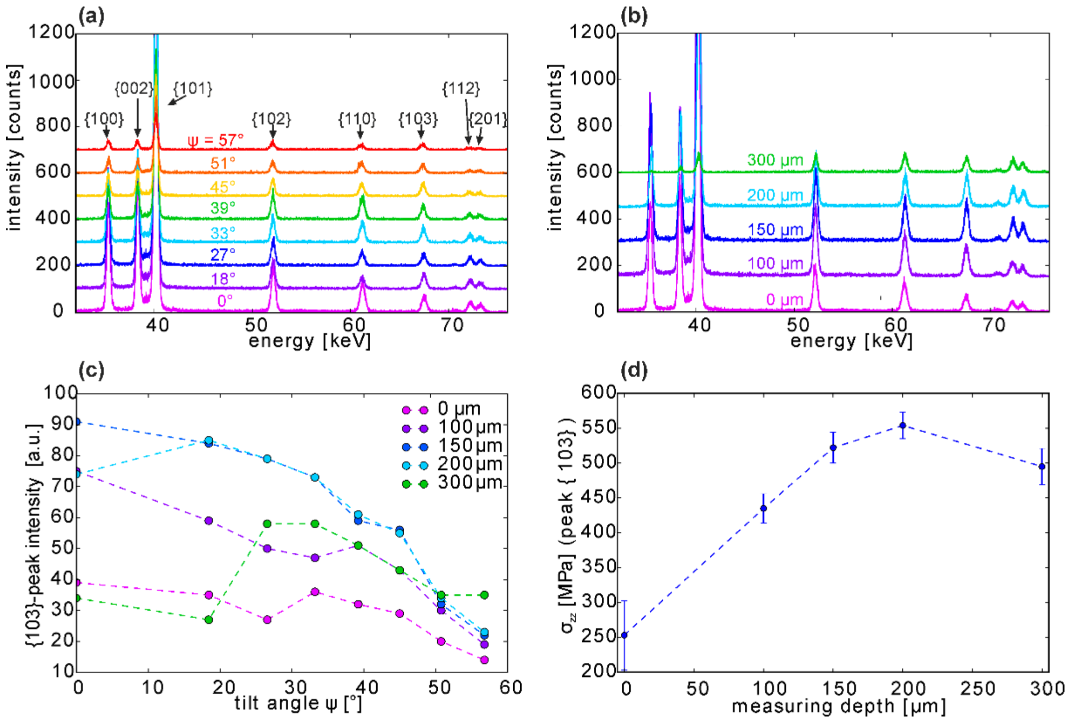

Figure 1. Diffractograms (**a**) for different ψ-angles (y = 0 μm measuring depth, ϕ = 0°); (**b**) for different measuring depths y (ψ = 0°, ϕ = 0°); (**c**) integrated {103}-peak intensities dependent on the measuring depths (ψ = 0°, ϕ = 0°); (**d**) RSs (residual stresses) in build direction σ_{zz} depending on the measuring depth. Note: the lines connecting the measurement points are only provided as a guide for the eye and do not represent actual physical relationships.

Additional Figures

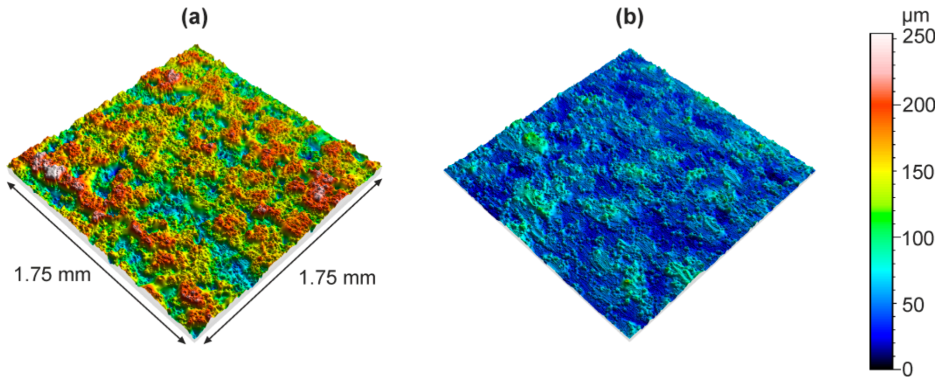

Figure 2. LSM topology data of selected samples: (a) specimen #04 (no contour lines, volume parameters P = 175 W, v = 500 mm/s) with the highest as-built roughness (Sa = 30 μm) and (b) specimen #19 (contour parameters Pcl = 300 W, vcl = 1575 mm/s) with the lowest as-built roughness (Sa = 13 μm) in this study.

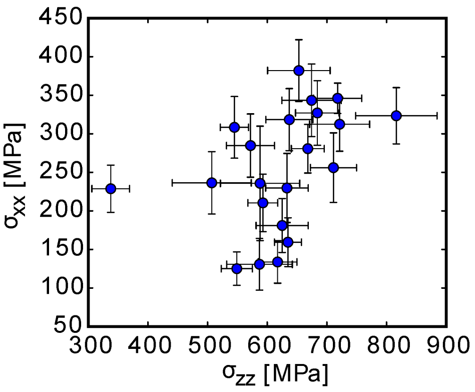

Figure 3. Analysis of potential correlations between stresses perpendicular to the build direction σ_{xx} and in build direction σ_{zz}. No specific trend with regard to scan stratgies, volume energy density etc. was recognized: high stresses in one direction did not always correlate with high values in the other (see Table S2 above).

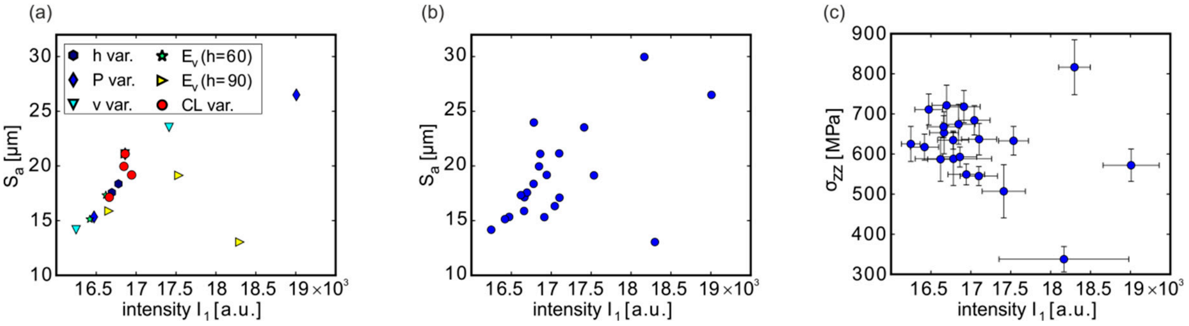

Figure 4. (a) comparison of S_a with I_1 for specimens which were manufactured with contour lines and the standard scan order CL(O-I)-V. (h var.: samples from the hatch distance variation; P var.: samples from the laser power variation; v var.: samples from the velocity variation; E_v (h = 60): samples from the E_v=const. study with hatch distance h = 60 µm; E_v (h = 90): samples from the E_v = const. study with hatch distance h = 90 µm; CL var.: samples from the contour line number variation). (b) comparison of S_a with I_1 for all specimens independent of a specific contour scan strategy. (c) Correlation between σ_{zz} and I_1 for all manufactured specimen.

For further discussion, please see the respective results in Fig. 19 of the main manuscript (Sect. 4.3).

Additional Tables

Table 1. Measured values: MPM data, surface roughness data and residual stress components.

Sample No	I_1 [a.u.]	I_2/I_1 [-]	S_a [μm]	S_z [μm]	σ_{zz} (y=150μm) [MPa]	Standard deviation σ_{zz} (y=150μm) [MPa]	σ_{xx} (y=150μm) [MPa]	Standard deviation σ_{xx} (y=150μm) [MPa]
#01	16664	1.0647	17.1	147.6	653	52	382	40
#02	17103	1.0765	17.1	159.6	637	39	318	40
#03	16782	1.0731	24.0	264.4	588	66	236	74
#04	18167	1.0966	30.0	254.0	338	32	229	31
#05	16862	1.0707	21.1	169.7	593	25	210	37
#06	17100	1.0792	21.1	184.5	545	24	308	40
#07	17044	1.0787	16.3	159.5	684	37	327	42
#08	16913	1.0719	15.3	166.4	718	40	346	20
#09	16943	1.0679	19.2	194.9	549	26	125	22
#10	16847	1.0700	20.0	171.3	674	50	343	47
#11	16473	1.0640	15.3	146.5	711	39	256	45
#12	19008	1.0706	26.5	221.0	572	40	285	41
#13	17412	1.0709	23.5	200.9	507	66	236	40
#14	16247	1.0612	14.2	159.8	625	44	181	35
#15	16778	1.0667	18.4	173.1	635	23	159	32
#16	16696	1.0667	17.6	178.9	721	50	312	35
#17	16659	1.0660	15.9	138.4	668	28	281	32
#18	17535	1.0643	19.1	159.7	633	36	230	45
#19	18299	1.0483	13.0	146.4	816	68	323	36
#20	16420	1.0655	15.1	159.4	617	32	134	28
#21	16620	1.0676	17.3	158.9	587	55	131	34

References

[1] Z. Chen, X. Wu, D. Tomus, C.H.J. Davies, Surface roughness of Selective Laser Melted Ti-6Al-4V alloy components, Add. Manuf. 21 (2018) 91-103.

[2] E.C. Oliver, M.R. Daymond, J. Quinta da Fonseca, P.J. Withers, Intergranular Stress Evolution in Titanium Studied by Neutron Diffraction and Self-consistent Modelling, J. Neutron Res. 12(1-3) (2004) 33-37.

[3] T. Mishurova, K. Artzt, J. Haubrich, G. Requena, G. Bruno, Exploring the Correlation between Subsurface Residual Stresses and Manufacturing Parameters in Laser Powder Bed Fused Ti-6Al-4V, Metals 9(2) (2019).

[4] T. Mishurova, K. Artzt, J. Haubrich, G. Requena, G. Bruno, New aspects about the search for the most relevant parameters optimizing SLM materials, Addit. Manuf. 25 (2019) 325-334.
